# Unveiling Genetic Markers for Milk Yield in Xinjiang Donkeys: A Genome-Wide Association Study and Kompetitive Allele-Specific PCR-Based Approach

**DOI:** 10.3390/ijms26072961

**Published:** 2025-03-25

**Authors:** Chao Fang, Frederic Farnir, Lingling Liu, Haixia Xiao

**Affiliations:** 1 College of Animal Science, Xinjiang Agricultural University, Urumqi 830052, China; 2 Faculté de Médecine Vétérinaire, Université de Liège Quartier Vallée, 2 Avenue de Cureghem, 6 (B43) 4000 Liège, Belgium; f.farnir@uliege.be; 3 Xinjiang Academy of Animal Sciences, Urumqi 830052, China

**Keywords:** GWAS, SNPs, Xinjiang donkey, milk, candidate gene

## Abstract

Lactation traits are critical economic attributes in domestic animals. This study investigates genetic markers and functional genes associated with lactation traits in Xinjiang donkeys. We analyzed 112 Xinjiang donkeys using 10× whole genome re-sequencing to obtain genome-wide single nucleotide polymorphisms (SNPs). Genome-wide association analyses were conducted using PLINK 2.0 and GEMMA 0.98.5 software, employing mixed linear models to assess several lactation traits: average monthly milk yield (AY), fat percentage (FP), protein percentage (PP), and lactose percentage (LP). A total of 236 SNPs were significantly associated with one or more milk production traits (*p* < 0.000001). While the two-software identified distinct SNP associations, they consistently detected the same 11, 95, 5, and 103 SNPs for AY, FP, PP, and LP, respectively. Several of these SNPs are located within potential candidate genes, including glycosylphosphatidylinositol anchored high density lipoprotein binding protein 1 (GPIHBP1), FLII actin remodeling protein (FLII), mitochondrial topoisomerase 1 (TOP1MT), thirty-eight-negative kinase 1 (TNK1), polo like kinase 1 (PLK1), notch homolog 1 (NOTCH1), developmentally regulated GTP-binding protein 2 (DRG2), mitochondrial elongation factor 2 (MIEF2), glutamine-fructose-6-phosphate transaminase 2 (GFPT2), and dual-specificity tyrosine phosphorylation-regulated kinase 2 (DYRK2). Additionally, we validated the polymorphism of 16 SNPs (10 genes) using Kompetitive Allele Specific PCR, revealing that TOP1MT_g.9133371T > C, GPIHBP1_g.38365122C > T, DRG2_g.4912631C > A, FLII_g.5046888C > T, and PLK1_g.23585377T > C were significantly correlated with average daily milk yield and total milk yield in the studied donkeys. This study represents the first genome-wide association analysis of markers and milk components in Xinjiang donkeys, offering valuable insights into the genetic mechanisms underlying milk production traits. Further research with larger sample sizes is essential to confirm these findings and identify potential causal genetic variants.

## 1. Introduction

Donkey milk has a lot of unsaturated fatty acids, especially linoleic acid, and low contents of fat and cholesterol, and is rich in calcium and selenium [1,2]. Donkey milk also has strong antioxidant activity, delaying the aging process, and is rich in kinds of immune-boosting substances [3]. It has emerged that donkey milk, compared with the milks of other animal species, is the nearest to human milk and an excellent substitute for it [4]. In some countries in Europe and America, donkey milk is not only the component of many biological products, but also a kind of healthcare product which is accepted by more and more people [5]. However, the commercial production of donkey milk is significantly limited by the species’ naturally low milk yield, leading to high production costs and insufficient supply. This challenge not only restricts the market potential of donkey milk as a functional food but also highlights the critical need to improve donkeys’ lactation performance [6].

Recently, significant progress has been achieved in understanding the genetic basis of lactation traits in livestock, driven by advanced molecular techniques like GWAS [7], RNA sequencing (RNA-seq) [8], and clustered regularly interspaced short palindromic repeats/CRISPR-associated protein 9 (CRISPR/Cas9) genome editing [9]. Key loci and genes have been firmly linked to milk yield and quality in dairy cattle and goats, including pyruvate carboxylase (PC) [10], diacylglycerol o-acyltransferase (DGAT) [11], peroxisome proliferator activated receptor gamma (PPARG) [12], and casein alpha s1 (CSN1S1) [13]. However, due to the unique physiological features and lactation processes in Equine species, it is yet to be established whether these known loci and genes are applicable to donkeys.

To date, numerous genes in donkeys have been studied using NGS, which have been found to be significantly associated with key phenotypic traits, including coat color variations (KIT ligand KITLG, t-box transcription factor 3 TBX3) [14], morphometric traits (cytochrome c oxidase subunit 1 COX 1, keratin 10 KRT10, keratin 1 KRT1, claudin 9 CLDN9, matrix metallopeptidase 28 MMP28) [15], carcass traits (NK1 homeobox 2) [16], body size (ligand-dependent nuclear receptor compression-like protein (LCORL)) [17], and growth traits (IGF2) [18]. Yu et al. identified threetarget genes (Decorin DCN, Integral Membrane Protein 2A ITM2A, and Arrestin Domain Containing 2 ARRDC2) associated with skeletal muscle growth and development [19]. However, few genes have been linked to milk traits in donkeys. He et al. demonstrated that NUMB endocytic adaptor protein (NUMB) g.46709914T > G could serve as a potential genetic marker for lactation traits [20]. During lactation, the fat and protein content in donkey milk decreases, whereas the lactose content increases-a phenomenon unique to donkeys compared to ruminants [21]. Therefore, conducting GWAS on donkey lactation performance based on SNPs seems to be great interest. Consequently, this study aimed to use GWAS to identify the loci involved in donkey milk traits. Additionally, we searched for candidate genes near the identified markers to establish a basis for future research aimed at uncovering the molecular mechanisms underlying these crucial lactation traits.

## 2. Results

### 2.1. Descriptive Statistics

The descriptive statistics of the phenotypic traits for the analyzed samples are summarized in Table 1. Xinjiang donkeys exhibit an average monthly milk yield of 38.51 kg, characterized by average compositions of 1.02% fat, 1.44% protein, and 6.46% lactose.

### 2.2. Resequencing of Xinjiang Donkey

We generated genomic sequences from the 112 Xinjiang donkeys, producing 2986.2 Gb of clean data with an average sequencing depth of 9.3× for subsequent analyses (Tables S1 and S2). On average, 96.26% (95.05–96.85%) of the reads were successfully mapped to the reference genome (Table S3).

Following SNP calling and quality control, we identified 12,039,909 high-quality SNPs across all 112 individuals. Based on NR annotation, the majority of SNPs were located in intergenic regions (5,201,198; 43.20%), followed by introns (4,738,320; 39.36%), upstream gene regions (898,713; 7.46%), and downstream gene regions (730,993; 6.07%) (Table S4).

### 2.3. Principal Component Analysis

**Figure 1:**
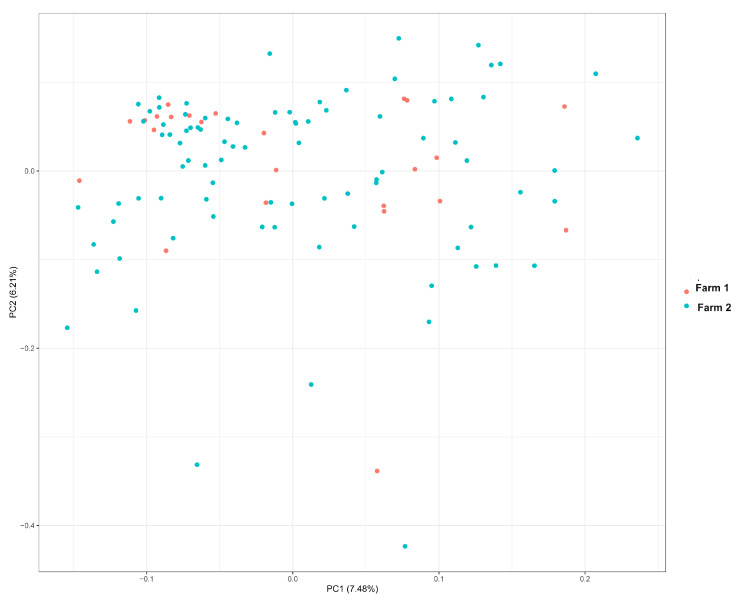
Population structure from the principal component analysis of the 112 Xinjiang donkeys.

In order to detect and correct for potential sub-structuration of the sample, we performed a principal component analysis (PCA) using PLINK 2.0. The animals involved in this study came from one breed and two breeding herds (farm 1 and farm 2). The PCA plot did not reveal obvious sub-structuration of the sample (Figure 1). Specifically, the first two principal components (PC1 and PC2) accounted for 7.48% and 6.21% of the variance, respectively.

### 2.4. GWAS Based on GEMMA and PLINK

**Figure 2:**
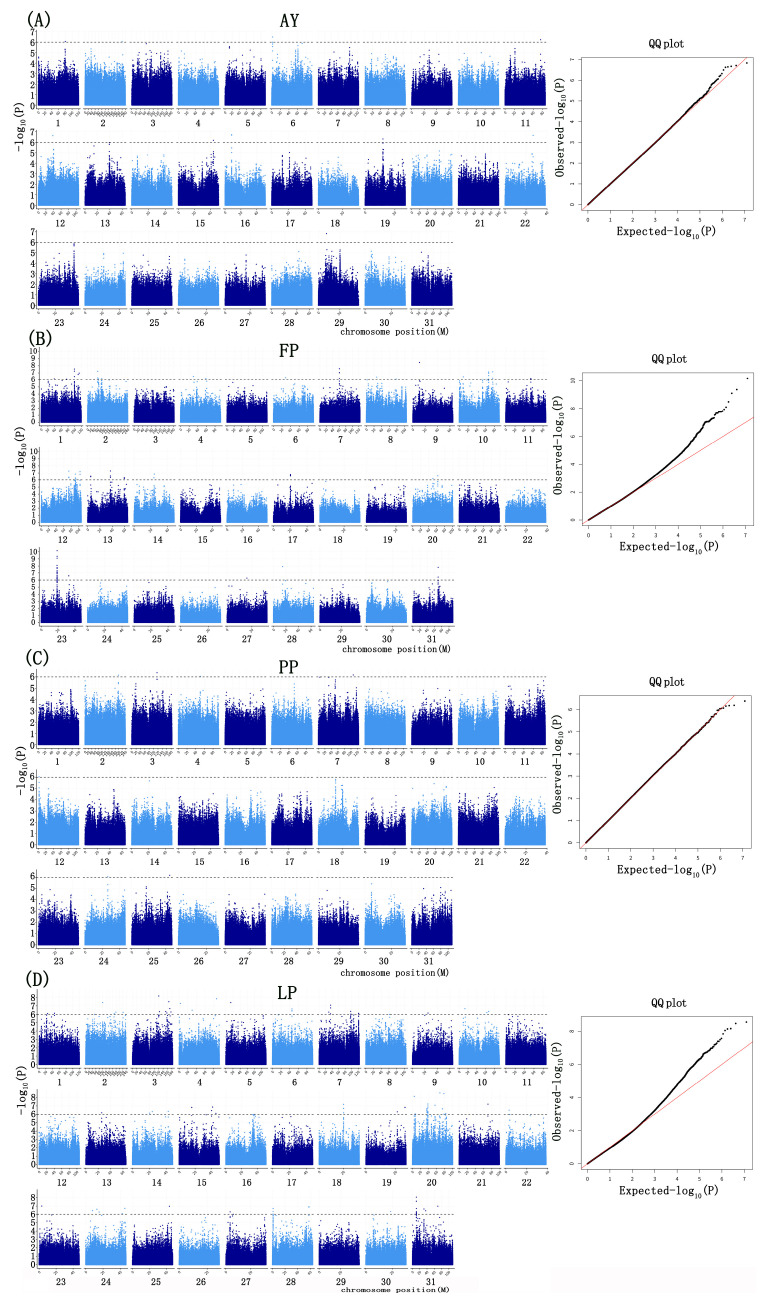
Manhattan of SNPs associated with the milk production traits based on GEMMA. (**A**) AY; (**B**) FP; (**C**) PP; (**D**) LP. Note: The red diagonal line typically represents the reference line where the theoretical distribution perfectly matches the actual data. The black dots represent the actual data points. If the data points approximately fall along a straight line, it indicates that the data follows a normal distribution.

**Figure 3:**
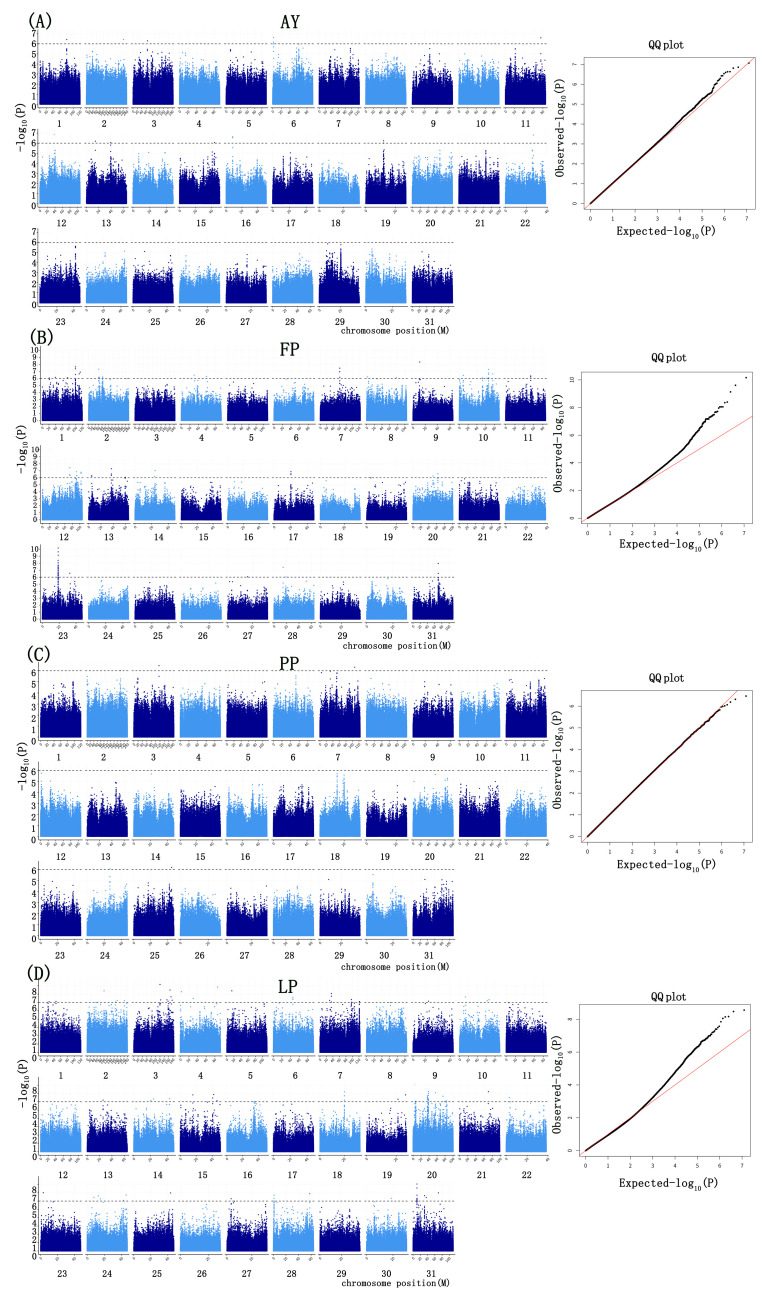
Manhattan of SNPs associated with the milk production traits based on PLINK. (**A**) AY; (**B**) FP; (**C**) PP; (**D**) LP.

For the four milk production-related phenotypes (AYs, FPs, PPs, LPs), significant SNPs were identified using both GEMMA and PLINK, with significance determined by the Bonferroni correction threshold (−log10 *p*-value ≥ 6) (Figure 2 and Figure 3).

**Figure 4:**
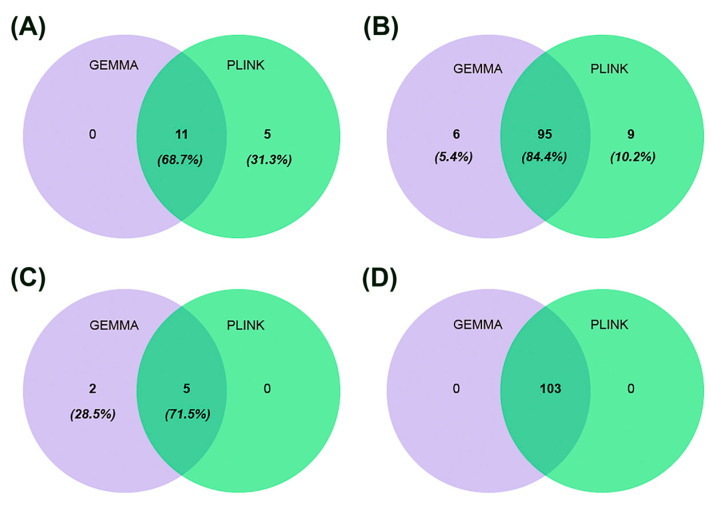
Prediction of SNPs for lactation traits based on GEMMA and PLINK. (**A**) SNPs for AY. (**B**) SNPs for FP. (**C**) SNPs for PP. (**D**) SNPs for LP.

Based on the GEMMA analysis, we identified 222 significant SNPs associated with four traits, distributed across 31 chromosomes. Of these SNPs, 11 were associated with AY, 101 with FP, 7 with PP, and 103 with LP, indicating that these markers were linked to only one trait. Similarly, using PLINK software, we identified 228 significant SNPs associated with the same four traits. Specifically, 16 SNPs were associated with AY, 104 with FP, 5 with PP, and 103 with LP, further confirming that these markers were linked to a single trait (Figure 4). In both GEMMA and PLINK analyses, 11 SNPs were found to be associated with the AY, 95 with the FP, 5 with the PP, and 103 with the LP (Figure 4).

### 2.5. Candidate Genes

**Figure 5:**
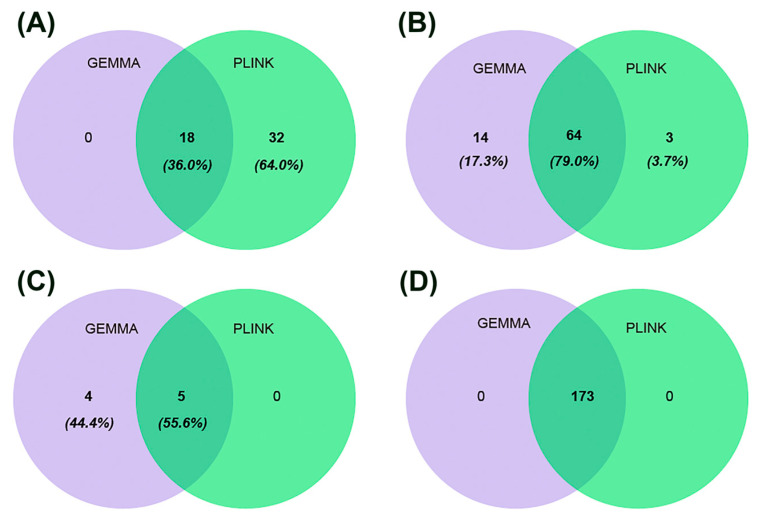
Prediction of candidate genes for lactation traits based on GEMMA and PLINK. (**A**) Genes for AY. (**B**) Genes for FP. (**C**) Genes for PP. (**D**) Genes for LP.

**Figure 6:**
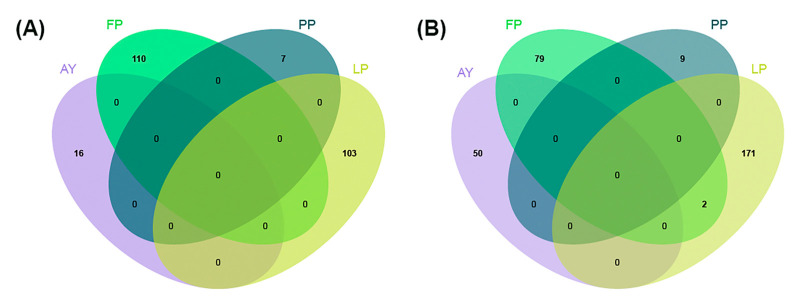
SNPs and candidate genes for four traits (AY, FP, PP, and LP) crossed over. (**A**) SNPs for four traits. (**B**) Candidate genes for four traits.

We annotated the genomic coordinates of all associated SNPs on the donkey genome using the NR database. We found that 60–70% genes (71.48% identified by GEMMA, 64.51% identified by PLINK) were unknown genes. For these four traits, GEMMA and PLINK identified largely overlapping sets of candidate genes. Specifically, both tools pinpointed the same gene sets for each trait: 18 for AY, 64 for FP, 5 for PP, and 173 for LP (Figure 5). In summary, we identified 16 SNPs associated with AY, 110 SNPs associated with FP, 7 SNPs associated with PP, and 103 SNPs associated with LP (Figure 6A). By combining results from GEMMA and PLINK, we found 50 genes linked to AY, 81 to FP, 9 to PP, and 173 to LP (Figure 6B). Notably, PLINK-predicted genes for the LP trait were also confirmed by GEMMA. Figure 6 shows that there is some overlap in candidate genes for specific phenotypic pairs, but no single gene is linked to three or four phenotypes simultaneously. For LP, two candidate genes were also associated with FP (such as, LOC106839422, calcineurin like EF-hand protein 2 CHP2). Our findings suggest that GPIHBP1, FLII, TOP1MT, and TNK1 could act as key regulatory genes for donkey milk yield. Likewise, peptidyl arginine deiminase 1/3 (PADI1/3), CHP2, GFPT2 and DYRK2 are probably the key genes for LP. For PP, cardiac α-actin 1 (ACTC1) and gap junction protein delta 2 (GJD2) are probably the key genes. Moreover, NOTCH1, DRG2, MIEF2 and PLK1 may be candidate genes affecting FP, which means that these could be the key genes affecting the lactation traits of donkeys.

### 2.6. Validation of Candidate Genes Using KASP Technology

#### 2.6.1. Locus Information of Candidate Genes

We identified 16 mutation sites in 10 candidate genes, based on GWAS findings, within the resequencing data. The detailed results can be found in Table 2.

#### 2.6.2. Genotyping Results by KASP

All 16 SNPs were polymorphic across the Xinjiang donkeys and further used for the association study.

#### 2.6.3. Polymorphism Analysis

The different genotype frequencies are shown in Table 3. The Fisher exact test of the SPSS 23 software was used to calculation χ^2^ and the *p*-value. Two SNPs (DRG2_g.4912631 C > A, TNK1_g. 14416183A > C) were not in HWE, which may be due to artificial selection.

#### 2.6.4. Correlation Analysis of Genotype and Milk Yield in Xinjiang Donkey

Eight SNPs significantly influenced the average daily and total milk yields of Xinjiang donkeys (Table 4). The TT of NOTCH1_g.9133371T > C was significantly higher than the CC average daily milk yield and total milk yield (*p* < 0.01); the AA of TOP1MT_g. 38323301G > A mutant increased daily milk yield and total milk yield (*p* < 0.01); The wild genotype CC of DRG2_g.4912631C > A yielded more milk than the mutant AA in both average daily and total milk yields, whereas the DRG2_g.4939345C > T mutation showed the opposite trend. The TT of FLII_g.5046888C > T mutant increased daily milk yield and total milk yield (*p* < 0.01); The wild genotype GG of PLK1_g.23587802G > C yielded more milk than the mutant CC, whereas the PLK1_g.23585377T > C mutation showed the opposite effect.

## 3. Discussion

### 3.1. Feasibility of GWAS Analysis Software

In this respect, GWASs have been proved to be powerful statistical analysis methods for detecting and identifying the genetic variation of complex and economically important traits. Several software are available to perform GWASs. In this study, we used two software: PLINK [22] and GEMMA [23,24]. PLINK is a freely available, widely used open-source toolset for genetic association that allows for the study of large datasets of genotypes and phenotypes. Genome-wide Efficient Mixed Model Association (GEMMA) can fit a univariate linear mixed model, a multivariate mixed model, and a Bayesian sparse linear mixed model for testing marker associations with a trait of interest in different organisms. One of the main challenges for GWAS analysis is managing false positives and false negatives that may occur due to population structure and familial relationships. To address this issue, mixed linear models (MLMs) are commonly used, incorporating covariates for structure and kinship to control for false positives [14]. PLINK has become the most popular software package for GWAS, PLINK operates much faster than MLM-based software [15], and GEMMA software was an efficient exact method that makes these approximations unnecessary in many settings [25].

### 3.2. Candidate Genes Related to Milk Yield (AY) in Xinjiang Donkeys

Milk is an important economic trait of cattle, horses, donkeys, camels, and other animals. Equine milk has higher edible and medicinal value than cow milk but equines produce significantly less milk than cattle. Under normal circumstances, the average monthly milk yield of dairy cows is between 500 and 1250 kg [26], while the average lactation of equine animals is about 30 kg per month [27,28]. In our study, the average monthly lactation of Xinjiang donkeys was 38.52 kg, which was a relatively good lactation performance for donkeys. Fifty candidate genes related to donkey milk yield are suggested by GWAS in two software, including TOP1MT, GPIHBP1, and TNK1. TOP1MTis an important mitochondrial DNA topoisomerase. In normal cells, TOP1MT deletion affects the expression of mitochondrial DNA and reduces important metabolic molecules in processes such as glucose oxidation and peptidation. It indicates a potential involvement in the mammary gland function. TNK1 belongs to the ACK (Activated Cdc42 Kinases) family of intracellular non- receptor tyrosine kinases that usually act as an important regulator in cytokine receptor-mediated intracellular signal transduction pathways [29]. One study showed that TNK1 modulates JAK-STAT signaling through phosphorylating STAT1 [30]. The JAK-STAT pathway regulates lactation [31]; furthermore, it has been illustrated that, by using the JAK-STAT pathway, the lactogenic hormones, through their receptors on cell membranes, regulate milk proteins [32]. It is another indication of a possible link with the milk production in donkeys. GPIHBP1plays an important role in the transport and localization of lipoprotein lipase (LPL) [33]. GPIHBP1 was identified as strongly associated with both fat percentage and protein yield traits [34,35].

### 3.3. Candidate Genes Related to Milk Composition (FP, PP, and LP) in Xinjiang Donkeys

Previous data suggest that donkey milk shares much similarity with mare milk in being low in total solids (8 to 10%) and protein (1.5 to 1.8%) and being high in lactose (6 to 7%) [36]. The level of fat in donkey milk ranges from 0.28% to 1.82% [37]. Our study showed that the milk fat percentage of Xinjiang donkeys was 1.02%. The milk protein percentage is 1.44%, and the lactose percentage is 6.46%, which seems to be lower than that of donkeys in other places (1.80% and 7.40%, respectively) [38].

Eighty-one candidate genes related to FP were identified suggested by the GWAS in two methods; of these, the function of twenty-six genes were known. A significant number of these genes have been linked to breast cancer, MANSC domain containing 1 (MANSC1) [39], PLK1 [40], family with sequence similarity 98 member A (FAM98A) [31], CHP2 [41], msh homeobox 1 (MSX1) [42], cytokine like 1 (CYTL1) [43], fibrinogen C domain containing 1 (FIBCD1) [44], hydroxyacid oxidase 1 (HAO1) [45], AlkB homolog 7 (ALKBH7) [46], DYRK2 [47], fatty acid binding protein 7 (FABP7) [48], etc. Uncoupling protein-1 (UCP1) plays a central role in energy dissipation in brown adipose tissue (BAT). Surprisingly, FLII acts as a regulatory complex for UCP1 transcription. So, we speculate that it may be related to the fat in donkey milk. Another notable transcription factor enriched for FP positional candidate genes was NOTCH1, which indicated the importance of the NOCTH signaling pathway in milk of Xinjiang donkey regulation. Do et al. identified that NOTCH1 might be important in the regulation of bovine milk cholesterol content [49]. DRG2 participates in the regulation of the proliferation and differentiation of multiple cells. It controls PPAR-g activity by interacting with PPAR-g. Strikingly, multiple GTPases, including GTP-binding proteins, are known to modulate adipogenesis by regulating PPAR-g expression [50,51]. Overall, DRG2 plays an active role in regulating adipocyte differentiation [52]. So, we speculate that it may participate in the production of milk fat. MIEF2 was one of the key regulators of mitochondrial fission [53]. One study demonstrated that MIEF2 significantly promoted lipid synthesis [54]. Nine candidate genes related to PP were identified by two methods, but the gene’s function was almost unknown, like LOC106835211, LOC123285010, and LOC123280491. Most studies report that the ACTC1 gene was associated with dilated cardiomyopathy [55,56]. Gap junctions of the insulin-producing β-cells are made of connexin 36 (Cx36), which is encoded by the GJD2 gene [57]. Signaling through gap junctions contributes to control insulin secretion and, thus, blood glucose levels. Li et al. found that candidate genes ACTC1 and GJD2 have putative roles in the regulation of mammary gland development [58].

A total of 173 candidate genes related to LP were identified. It has been reported that PADI3 (Protein Arginine Deiminase 3) may act on glucose metabolism through COPII and PKM pathways, which may greatly affect lactose yield [59]. DYRK2, a member of the class II DYRK family protein, was a key regulator of p53, and phosphorylates it at Ser46 to induce apoptosis in response to DNA damage [60]. According to previous research, they found that DYRK2 was closely associated with milk traits in Xinjiang Brown cattle [61]. The 11 bp InDel in DYRK2 that Mao et al. identified was significantly correlated with milk traits in Xinjiang Brown cattle [62]. Fatty acid-binding protein 7 (FABP7) involved in intracellular lipid dynamics. The overexpression of FABP7 gene prevents ATP production from glucose [63].

## 4. Materials and Methods

### 4.1. Animals and Phenotyping

In total, 4704 test-day records were collected from 112 Xinjiang donkeys born from 2013 to 2014 and reared in 2 donkey farms in Yopurga county, Kashgar region. Four milk production traits (average monthly milk yield (AY), fat percentage (FP), protein percentage (PP), and lactose percentage (LP)) were recorded. In addition, we selected 82 Xinjiang donkeys as a validation population to collect daily milk yield and milk yield throughout lactation.

### 4.2. Blood Sample Collection and DNA Extraction

A total of 5 mL blood of Xinjiang donkey were collected from jugular vein and anticoagulated with EDTA; 200 μL of genomic DNA were extracted using Animal Blood/Cell/Tissue Genomic DNA Extraction Kit (Tiangen Biochemical Technology company, Beijing, China, DP304-03).

### 4.3. Monitoring of Genomic DNA

Three methods were used to detect DNA: (1) Agarose gel electrophoresis for DNA purity and integrity; (2) Nanodrop detection of DNA purity (OD 260/280 ratio); (3) Qubit 2.0 for accurate quantification of DNA concentration.

### 4.4. Library Construction

The Illumina whole genomes of 112 donkey individuals were sequenced from Novogene company. The high-quality genomic DNA samples were randomly interrupted into 350 bp using a Covaris ultrasonic fragmentation instrument (LE220R-plus), followed by end repairing, the addition of A-tail, the addition of a sequencing adaptor, purification, and PCR amplification steps. After library construction, initial quantification was performed using Qubit 2.0 to dilute the library, followed by the detection of the insert fragments of the library using Agilent 2100 (Agilent Technologies, Santa Clara, CA, USA, G2939A). Sequencing was performed using Illumina high-throughput sequencing platform NovaSeq 6000 (Illumina, San Diego, CA, USA).

### 4.5. Variant Sites Detection

Quality control for markers was performed using the Fastp (0.19.7) software. The steps of data processing were as follows: (1) Discard a paired reads if either read contains adapter contamination; (2) Discard a paired reads if more than 10% of bases are uncertain in either read; (3) Discard paired reads if the proportion of low-quality (Phred quality < 5) bases is over 50% in either read. Clean data were aligned to the donkey reference genome (Equus asinus [64]) using BWA (0.7.17) software [65] and PCR duplicates were removed using Picard’s Mark Duplicate tool (http://sourceforge.net/projects/picard/, accessed on 5 April 2023). The detection of SNP variant loci was completed using GATK haplotype-caller [66].

### 4.6. Variant Sites Filtering

We used PLINK to perform SNPs filtering with the following inclusion criteria: (1) SNP call rate > 80%; (2) Hardy–Weinberg equilibrium *p*–value > 0.01; and (3) minor allele frequency (MAF) > 0.05.

### 4.7. Genome-Wide Association Study

Genome-wide association analysis was performed using a mixed-model approach with two software (GEMMA, PLINK). We did not include individual additive random effects. We used the following statistical model:

y = W × α + Xs × βs + g + e (1)

In this equation, the following definitions apply:

y is an n × 1 vector of phenotypic values (phenotypes are either AY, FP, PP, or LP; n = 112);

W is an n × c incidence matrix associating the fixed effects to the corresponding phenotypes;

α is a c × 1 vector of the fixed effects, including an overall mean, the parity (either 5 or 6), first two PCA components and the birth year of the donkey (either 2013 or 2014); consequently, c = 7;

Xs is an n × 1 vector of SNP genotypes (coded as 0, 1 or 2, where 0 and 2 correspond to the homozygous and 1 to the heterozygous genotypes) at the tested position;

βs is the allelic substitution effect for the tested marker;

g is a n × 1 vector of random polygenic effects distributed as $g\sim N\left(0,\sigma_{A}^{2}*\varphi \right)$, where $\sigma_{A}^{2}$ is the additive genetic variance and φ is the relationship matrix, using the same methodology described by van Randen [67];

e is an n × 1 vector of residual errors with $e\sim N\left(0,\sigma_{e}^{2}*I\right)$, where $\sigma_{e}^{2}$ is the residual variance and I is an n × n identity matrix.

The thresholds of the Bonferroni-corrected *p*-values for suggestive genome-wide significance associations were set as 1 × 10^−6^.

### 4.8. KASP

Competitive Allele Specific PCR (KASP^TM^, LGC Genomics, Teddington, Middlesex, UK) genotyping was used for the biallelic discrimination of the 16 selected SNPs. Genotype data for 82 Xinjiang donkeys were exported for statistical analysis.

### 4.9. Primer Design and PCR

Prime premier 6.0 was used to design the primer of 10 genes. Primer sequences are given in Table 5. PCR amplification was conducted in a 10 μL volume containing 2 × Master Mix 2.5 μL, 100 μM primer 0.03 μL, 100 μM Primer Common 0.04 μL, genomic DNA 2 μL, and ddH_2_O supplement 5.43 μL. The PCR conditions were as follows: an initial step at 94 °C (one cycle for 10 min), 10 cycles for 20 s at 94 °C, 61 s at 45 °C for each primer pair, and 20 cycles for 20 s at 94 °C, 20 s at 55 °C.

### 4.10. Statistical Analysis

The chi-squared test (χ^2^) was used to determine whether the populations were in Hardy–Weinberg equilibrium (HWE). One-way ANOVA was used to study the genetic association with polymorphic SNPs by the software SPSS 23. Duncan’s method was used to multiple comparisons. Data are expressed as the means ± SE.

## 5. Conclusions

This is the first reported GWAS for milk traits in Xinjiang donkeys. In the current study, we detected 236 SNPs associated with one of four milk production traits (AY, FP, PP, and LP). These SNPs are distributed on the 1–31 chromosomes of donkeys, and some of them are placed within or close to potential candidate genes. The consistence of our identified genomic regions with candidate genes provides further evidence for the importance of these candidate genes for the variation in milk production traits. The validation of 16 SNPs using Kompetitive Allele Specific PCR further confirmed the significant correlation of specific variants with milk yield. Further confirmation studies including a larger population size should be performed to validate the findings and potentially identify the causal genetic variants.

## Figures and Tables

**Table 1:** Descriptive statistics of the milk production traits in Xinjiang donkey.

Traits	n	Mean	SD	Minimum	Maximum
AY (kg)	112	38.51	14.87	13.60	72.75
FP (%)	112	1.02	0.93	0.12	4.52
PP (%)	112	1.44	0.19	0.99	1.94
LP (%)	112	6.46	0.39	4.47	6.96

Note: AY is obtained by calculating the average daily milk yield based on the test-day milk and multiplying it by 30.

**Table 2:** Selected SNPs used in the study for genotyping the Xinjiang donkey.

Official Full Name of Gene	Gene	Gene ID	Allele Substitution	Position	Chromosome
Glutamine-fructose-6-phosphate transaminase 2	*GFPT2*	106843727	C/A	25060895	9
	*GFPT2*	106843727	T/G	25062599	9
Notch receptor 1	*NOTCH1*	106845559	T/C	9133371	10
DNA topoisomerase I mitochondrial	*TOP1MT*	106841874	G/A	38323301	12
Glycosylphosphatidylinositol anchored high density lipoprotein binding protein 1	*GPIHBP1*	106841943	C/T	38365122	12
Developmentally regulated GTP binding protein 2	*DRG2*	106837927	C/A	4912631	13
	*DRG2*	106837927	C/T	4939345	13
FLII actin remodeling protein	*FLII*	106837933	G/C	5044716	13
	*FLII*	106837933	C/T	5046888	13
Mitochondrial elongation factor 2	*MIEF2*	106837926	G/T	5060681	13
Tyrosine kinase non receptor 1	*TNK1*	106844191	A/G	14413548	13
	*TNK1*	106844191	A/C	14416183	13
Polo like kinase 1	*PLK1*	106845827	T/C	23585377	14
	*PLK1*	106845827	G/C	23587802	14
Dual specificity tyrosine phosphorylation regulated kinase 2	*DYRK2*	106843596	C/G	3170005	22
	*DYRK2*	106843596	T/C	3171414	22

**Table 3:** List of polymorphic SNPs and genotypes frequencies in Xinjiang donkey.

Gene	Position	Allele Substitution	Genotype	Genotype Frequencies	χ^2^	*p*
*GFPT2*	25060895	C>A	CC	0.013	0.295	0.587
			CA	0.150		
			AA	0.837		
*GFPT2*	25062599	T>G	TT	0.063	1.902	0.348
			TG	0.225		
			GG	0.712		
*NOTCH1*	9133371	T>C	TT	0.288	1.618	0.472
			TC	0.400		
			CC	0.312		
*TOP1MT*	38323301	G>A	GG	0.500	3.005	0.232
			GA	0.325		
			AA	0.175		
*GPIHBP1*	38365122	C>T	CC	0.513	2.023	0.389
			CT	0.337		
			TT	0.150		
*DRG2*	4912631	C>A	CC	0.473	14.006	0.001
			CA	0.203		
			AA	0.324		
*DRG2*	4939345	C>T	CC	0.400	3.453	0.188
			CT	0.350		
			TT	0.250		
*FLII*	5044716	G>C	GG	0.950	1.450	0.719
			GC	0.038		
			CC	0.012		
*FLII*	5046888	C>T	CC	0.357	4.644	0.105
			CT	0.314		
			TT	0.329		
*MIEF2*	5060681	G>T	GG	0.738	2.236	0.338
			GT	0.200		
			TT	0.062		
*TNK1*	14413548	A>G	AA	0.014	0.290	1.000
			AG	0.043		
			GG	0.943		
*TNK1*	14416183	A>C	AA	0.792	35.321	<0.001
			AC	0.013		
			CC	0.195		
*PLK1*	23585377	T>C	TT	0.571	2.311	0.330
			TC	0.300		
			CC	0.129		
*PLK1*	23587802	G>C	GG	0.164	3.528	0.177
			GC	0.288		
			CC	0.548		
*DYRK2*	3170005	C>G	CC	0.914	1.293	0.764
			CG	0.071		
			GG	0.015		
*DYRK2*	3171414	T>C	TT	0.914	<0.001	1.000
			TC	0.086		
			CC	0		

**Table 4:** List of SNPs found to be associated with milk yield in Xinjiang donkey.

Gene	Position	Genotype	Average Daily Milk Yield (kg)	Total Milk Yield (kg)
*NOTCH1*	9133371	TT (23)	2.67 ± 0.15 ^Aa^	482.33 ± 28.02 ^Aa^
		TC (32)	2.42 ± 0.10 ^Aa^	435.89 ± 18.51 ^Aa^
		CC (25)	1.57 ± 0.19 ^Bb^	254.48 ± 35.16 ^Bb^
*TOP1MT*	38323301	GG (40)	1.86 ± 0.12 ^Bb^	316.47 ± 24.11 ^Bb^
		GA (26)	2.60 ± 0.16 ^Aa^	467.93 ± 28.16 ^Aa^
		AA (14)	2.61 ± 0.23 ^Aa^	469.92 ± 41.61 ^Aa^
*GPIHBP1*	38365122	CC (41)	1.88 ± 0.14 ^Bb^	320.12 ± 26.65 ^Bb^
		CT (27)	2.75 ± 0.13 ^Aa^	495.25 ± 22.99 ^Aa^
		TT (12)	2.27 ± 0.19 ^ABab^	408.95 ± 35.05 ^ABab^
*DRG2*	4912631	CC (35)	2.57 ± 0.12 ^Aa^	462.09 ± 21.32 ^Aa^
		CA (15)	1.98 ± 0.21 ^ABa^	345.35 ± 41.44 ^ABa^
		AA (24)	1.76 ± 0.18 ^Bb^	293.94 ± 33.67 ^Bb^
*DRG2*	4939345	CC (32)	1.74 ± 0.17 ^Bb^	291.96 ± 31.66 ^Bb^
		CT (28)	2.42 ± 0.10 ^Aa^	435.73 ± 18.53 ^Aa^
		TT (20)	2.74 ± 0.17 ^Aa^	493.05 ± 31.19 ^Aa^
*FLII*	5046888	CC (25)	1.66 ± 0.18 ^Bb^	270.49 ± 34.51 ^Bb^
		CT (22)	2.67 ± 0.13 ^Aa^	480.44 ± 24.28 ^Aa^
		TT (23)	2.51 ± 0.17 ^Aa^	451.18 ± 31.33 ^Aa^
*PLK1*	23585377	TT (40)	1.93 ± 0.15 ^Ab^	330.86 ± 29.08 ^Bb^
		TC (21)	2.65 ± 0.14 ^Aa^	476.60 ± 25.39 ^ABa^
		CC (9)	2.74 ± 0.23 ^Aa^	493.45 ± 41.05 ^Aa^
*PLK1*	23587802	GG (12)	2.62 ± 0.18 ^Aa^	471.73 ± 32.51 ^Aa^
		GC (21)	2.65 ± 0.14 ^Aa^	476.60 ± 25.39 ^Aa^
		CC (40)	1.93 ± 0.15 ^Ab^	330.86 ± 29.08 ^Bb^

Note: Different capital letters indicate extremely significant differences (*p* < 0.01), while different lowercase letters indicate significant differences (*p* < 0.05), comparison between different genotypes of a gene.

**Table 5:** Primer information.

Gene	Position	Primer Sequence
*GFPT2*	25060895	F1: GAAGGTCGGAGTCAACGGATTTCACATGGTCTCTCCTCCCAC
		F2: GAAGGTGACCAAGTTCATGCTTCACATGGTCTCTCCTCCCAA
		R: CCTTCATGGGGATTCACTGC
*GFPT2*	25062599	F1: GAAGGTCGGAGTCAACGGATTGGCTCGGCCTCCTGCTA
		F2: GAAGGTGACCAAGTTCATGCTGGCTCGGCCTCCTGCTC
		R: GCTGAGGCTCCGGGCTAT
*NOTCH1*	9133371	F1: GAAGGTCGGAGTCAACGGATTGATTGTCCTGCTGTTCAAACAC
		F2: GAAGGTGACCAAGTTCATGCTGATTGTCCTGCTGTTCAAACAT
		R: GCACTGCCCCCTCGC
*TOP1MT*	38323301	F1: GAAGGTCGGAGTCAACGGATTGGCGAAGACTTTGAGTTCTAAACA
		F2: GAAGGTGACCAAGTTCATGCTGCGAAGACTTTGAGTTCTAAACG
		R: ATAAACTCCAGTGAGGCACGAG
*GPIHBP1*	38365122	F1: GAAGGTCGGAGTCAACGGATTGCCGCAGGACAGGACAT
		F2: GAAGGTGACCAAGTTCATGCTGCCGCAGGACAGGACAC
		R: TGCCTCCCGCATTCTTC
*DRG2*	4912631	F1: GAAGGTCGGAGTCAACGGATTGCCTCTCTACTCGGTCACCG
		F2: GAAGGTGACCAAGTTCATGCTGGCCTCTCTACTCGGTCACCT
		R: GCCCCTGAGGGATCTGG
*DRG2*	4939345	F1: GAAGGTCGGAGTCAACGGATTGGCCTGGGGAAGGCA
		F2: GAAGGTGACCAAGTTCATGCTGGCCTGGGGAAGGCG
		R: CTCTCCTCCTGCAGCTCTCA
*FLII*	5044716	F1: GAAGGTCGGAGTCAACGGATTCGTGCAGGTACCCCCAG
		F2: GAAGGTGACCAAGTTCATGCTCGTGCAGGTACCCCCAC
		R: GCTGGGCATGACATGAGG
*FLII*	5046888	F1: GAAGGTCGGAGTCAACGGATTGCGCTGCCCTAGGCC
		F2: GAAGGTGACCAAGTTCATGCTGGCGCTGCCCTAGGCT
		R: CTGCTTCCATGCCTGGG
*MIEF2*	5060681	F1: GAAGGTCGGAGTCAACGGATTCACCCGTTCCGAGGCA
		F2: GAAGGTGACCAAGTTCATGCTCACCCGTTCCGAGGCC
		R: CACTGGGTCACACCATTCACA
*TNK1*	14413548	F1: GAAGGTCGGAGTCAACGGATTACAGGGGAAGGGAGGTTTT
		F2: GAAGGTGACCAAGTTCATGCTACAGGGGAAGGGAGGTTTC
		R: CCAGGCCGGACCCTG
*TNK1*	14416183	F1: GAAGGTCGGAGTCAACGGATTTCTGCTTCTTCACCTGGGG
		F2: GAAGGTGACCAAGTTCATGCTGTCTGCTTCTTCACCTGGGT
		R: ACTGTGCCAGGTTCCGG
*PLK1*	23585377	F1: GAAGGTCGGAGTCAACGGATTGAGAGTTCCCAGGAGGCAAT
		F2: GAAGGTGACCAAGTTCATGCTGAGAGTTCCCAGGAGGCAAC
		R: AGTCCCTGTCCACAGGTGG
*PLK1*	23587802	F1: GAAGGTCGGAGTCAACGGATTCAAACTCATCCTGTGCCCG
		F2: GAAGGTGACCAAGTTCATGCTCAAACTCATCCTGTGCCCC
		R: CGATGTAGGTCACGGCTGC
*DYRK2*	3170005	F1: GAAGGTCGGAGTCAACGGATTTGGGAAGGCAAGGTAATTATATG
		F2: GAAGGTGACCAAGTTCATGCTTGGGAAGGCAAGGTAATTATATC
		R: GGATTACATTTGCAATTATGTATCTG
*DYRK2*	3171414	F1: GAAGGTCGGAGTCAACGGATTTGCATCTCCAGGAAGGTCAG
		F2: GAAGGTGACCAAGTTCATGCTTGCATCTCCAGGAAGGTCAA
		R: GGAGGAGTAAATATTAATTACTTGGTTT

## Data Availability

Data are contained within the article and Supplementary Materials.

## Supplementary Materials

The following supporting information can be downloaded at: https://www.mdpi.com/article/10.3390/ijms26072961/s1.
