# Supplementary material for: Unveiling Genetic Markers for Milk Yield in Xinjiang Donkeys: A Genome-Wide Association Study and Kompetitive Allele-Specific PCR-Based Approach
Source: Int J Mol Sci. 2025 Mar 25;26(7):2961. doi: 10.3390/ijms26072961 (PMC11988640; doi:10.3390/ijms26072961)
Supplement: Supplementary file 1 [file ijms-26-02961-s001.zip › Table S1.pdf]

Table S1 Statistics of sequencing data evaluation

samples	Raw Reads	Clean Reads	Raw Base(G)	Clean Base(G)	Effective Rate (%)	Q20(%)	Q30(%)	GC Content (%)
14	88,759,801	88,178,739	26.63	26.45	99.35	97.53	92.96	40.78
109	84,811,483	84,339,755	25.44	25.3	99.44	97.15	92.13	41.43
78	100,568,616	99,966,324	30.17	29.99	99.40	97.55	92.96	41.31
110	87,980,458	87,479,588	26.39	26.24	99.43	97.06	91.94	41.09
55	86,243,428	85,673,080	25.87	25.7	99.34	97.53	92.97	41.03
59	87,580,728	87,087,850	26.27	26.13	99.44	97.50	92.84	40.43
85	90,509,573	89,917,438	27.15	26.98	99.35	97.43	92.70	41.70
45	87,713,122	87,248,117	26.31	26.17	99.47	97.50	92.82	40.42
82	85,612,024	85,122,261	25.68	25.54	99.43	97.57	92.99	40.45
4	97,832,135	97,176,562	29.35	29.15	99.33	97.37	92.63	42.51
2	91,718,426	91,196,972	27.52	27.36	99.43	97.40	92.66	40.89
77	85,861,786	85,275,063	25.76	25.58	99.32	97.47	92.84	42.71
50	90,735,019	90,223,531	27.22	27.07	99.44	97.36	92.61	41.52
10	95,627,225	94,993,333	28.69	28.5	99.34	97.42	92.75	41.81
21	84,804,910	84,324,888	25.44	25.3	99.43	97.44	92.72	41.01
3	88,375,517	87,820,112	26.51	26.35	99.37	97.53	92.93	41.18
39	96,677,246	96,121,296	29.0	28.84	99.42	97.53	92.97	40.74
40	86,231,559	85,748,095	25.87	25.72	99.44	97.53	92.91	40.59
60	86,132,766	85,573,307	25.84	25.67	99.35	97.47	92.79	40.59
65	90,723,397	90,138,155	27.22	27.04	99.35	97.03	91.90	42.38
105	90,466,957	89,979,796	27.14	26.99	99.46	97.34	92.54	40.50
23	91,085,506	90,543,568	27.33	27.16	99.41	97.53	92.95	41.50
20	84,031,785	83,518,811	25.21	25.06	99.39	97.30	92.43	42.09
8	87,957,030	87,349,029	26.39	26.2	99.31	97.41	92.75	41.67
102	93,942,371	93,202,720	28.18	27.96	99.21	96.82	91.57	42.08
61	94,250,137	93,711,842	28.28	28.11	99.43	97.46	92.84	41.50
44	85,969,186	85,440,948	25.79	25.63	99.39	97.53	92.96	40.70
80	85,565,241	85,010,500	25.67	25.5	99.35	97.53	92.91	41.02
104	88,602,627	87,982,111	26.58	26.39	99.30	97.35	92.66	41.32
5	94,735,292	94,066,184	28.42	28.22	99.29	97.48	92.96	43.50
27	101,186,965	100,634,160	30.36	30.19	99.45	97.52	92.94	41.12
24	85,174,101	84,641,006	25.55	25.39	99.37	97.27	92.40	41.24
6	89,171,540	88,557,765	26.75	26.57	99.31	97.42	92.75	41.56
1	85,785,893	85,277,024	25.74	25.58	99.41	97.31	92.45	41.00
56	88,428,192	87,906,138	26.53	26.37	99.41	97.51	92.89	40.45
30	86,470,662	85,983,061	25.94	25.79	99.44	97.45	92.78	40.75
29	117,113,284	116,450,480	35.13	34.94	99.43	97.47	92.78	41.04
53	86,277,512	85,785,884	25.88	25.74	99.43	97.34	92.51	41.41
48	92,282,697	91,673,550	27.68	27.5	99.34	97.56	93.04	40.48
94	87,216,535	86,704,842	26.16	26.01	99.41	97.43	92.66	40.61

46	85,068,632	84,628,600	25.52	25.39	99.48	97.51	92.84	40.46
52	87,068,282	86,494,405	26.12	25.95	99.34	97.28	92.41	41.58
33	87,858,139	87,404,759	26.36	26.22	99.48	97.13	92.12	40.72
37	98,875,940	98,333,761	29.66	29.5	99.45	97.53	92.90	40.73
69	85,644,813	85,124,479	25.69	25.54	99.39	97.48	92.83	41.03
95	86,661,951	86,145,237	26.0	25.84	99.40	97.53	92.94	41.22
76	86,554,027	86,047,540	25.97	25.81	99.41	97.59	93.08	41.22
43	89,760,561	89,190,366	26.93	26.76	99.36	97.19	92.21	40.80
96	86,428,028	85,864,409	25.93	25.76	99.35	97.38	92.69	40.53
79	85,837,162	85,290,463	25.75	25.59	99.36	97.55	92.91	40.53
86	84,026,616	83,521,904	25.21	25.06	99.40	97.61	93.11	41.02
41	87,810,821	87,294,680	26.34	26.19	99.41	97.48	92.84	41.80
42	90,774,990	90,177,142	27.23	27.05	99.34	97.55	93.05	40.79
57	85,352,755	84,906,164	25.61	25.47	99.48	97.43	92.70	41.26
108	88,459,036	87,899,982	26.54	26.37	99.37	97.17	92.20	40.75
49	89,205,385	88,720,248	26.76	26.62	99.46	97.38	92.57	40.48
73	94,625,913	94,035,555	28.39	28.21	99.38	97.46	92.78	41.51
67	85,016,256	84,652,567	25.5	25.4	99.57	97.00	91.77	40.35
38	99,778,482	99,183,742	29.93	29.76	99.40	97.60	93.09	40.80
70	86,826,705	86,297,562	26.05	25.89	99.39	97.54	92.92	40.69
28	91,866,925	91,289,539	27.56	27.39	99.37	97.47	92.81	41.03
35	83,568,093	83,174,195	25.07	24.95	99.53	96.84	91.44	40.80
101	85,845,185	85,340,333	25.75	25.6	99.41	97.35	92.61	40.53
100	87,371,282	86,843,476	26.21	26.05	99.40	97.20	92.31	41.61
15	87,715,969	87,180,801	26.31	26.15	99.39	97.31	92.53	41.91
98	84,699,907	84,165,421	25.41	25.25	99.37	97.47	92.88	41.03
111	93,358,694	92,774,948	28.01	27.83	99.37	97.08	92.00	41.11
66	85,900,565	85,480,696	25.77	25.64	99.51	97.00	91.78	41.08
7	89,565,595	88,911,147	26.87	26.67	99.27	97.45	92.79	42.51
31	87,205,799	86,803,535	26.16	26.04	99.54	97.08	91.94	41.20
92	89,962,365	89,407,254	26.99	26.82	99.38	97.53	92.86	40.38
47	94,046,591	93,504,868	28.21	28.05	99.42	97.54	92.97	40.77
97	91,279,950	90,739,659	27.38	27.22	99.41	97.51	92.99	41.12
81	87,368,924	86,805,877	26.21	26.04	99.36	97.48	92.83	41.50
106	86,259,941	85,804,815	25.88	25.74	99.47	97.12	92.06	40.48
71	99,141,793	98,589,920	29.74	29.58	99.44	97.45	92.71	40.84
54	89,263,248	88,735,013	26.78	26.62	99.41	97.55	93.00	40.55
32	86,422,093	85,951,616	25.93	25.79	99.46	97.06	91.89	40.99
75	91,717,464	91,184,941	27.52	27.36	99.42	97.65	93.19	40.95
62	98,351,145	97,699,199	29.51	29.31	99.34	97.43	92.78	42.66
11	88,214,101	87,622,931	26.46	26.29	99.33	97.21	92.29	42.05
84	85,636,805	85,163,960	25.69	25.55	99.45	97.56	92.94	40.30
99	88,160,560	87,578,233	26.45	26.27	99.34	97.43	92.82	41.30
90	95,004,121	94,494,268	28.5	28.35	99.46	97.34	92.45	41.09

103	88,138,293	87,581,968	26.44	26.27	99.37	97.30	92.50	41.05
12	83,645,063	83,181,859	25.09	24.95	99.45	97.42	92.69	41.17
68	90,962,504	90,529,153	27.29	27.16	99.52	96.88	91.47	40.62
17	86,903,640	86,398,647	26.07	25.92	99.42	97.16	92.13	41.55
93	84,638,237	84,166,865	25.39	25.25	99.44	97.30	92.38	41.11
26	86,693,458	86,218,654	26.01	25.87	99.45	97.25	92.30	40.78
16	87,359,694	86,843,243	26.21	26.05	99.41	97.55	93.00	41.61
107	89,972,982	89,463,327	26.99	26.84	99.43	96.91	91.59	40.44
64	91,697,855	91,018,466	27.51	27.31	99.26	96.97	91.89	43.88
19	87,910,593	87,355,677	26.37	26.21	99.37	96.78	91.31	41.23
51	86,335,365	85,793,936	25.9	25.74	99.37	97.38	92.67	42.19
83	99,536,931	98,929,189	29.86	29.68	99.39	97.52	92.88	40.48
112	89,861,128	89,291,230	26.96	26.79	99.37	97.14	92.13	41.09
72	84,442,409	84,009,410	25.33	25.2	99.49	97.72	93.28	40.19
89	86,042,366	85,536,622	25.81	25.66	99.41	97.35	92.48	40.96
25	88,943,801	88,454,687	26.68	26.54	99.45	96.78	91.21	41.03
22	88,256,410	87,754,664	26.48	26.33	99.43	97.38	92.66	41.46
74	96,409,240	95,821,439	28.92	28.75	99.39	97.50	92.89	41.64
13	88,450,822	87,828,740	26.54	26.35	99.30	97.27	92.38	41.95
58	83,455,675	82,969,792	25.04	24.89	99.42	97.43	92.69	40.34
18	93,049,153	92,444,462	27.91	27.73	99.35	97.46	92.84	41.64
9	87,934,008	87,385,148	26.38	26.22	99.38	97.30	92.44	41.25
36	88,582,124	88,163,779	26.57	26.45	99.53	96.91	91.59	40.72
88	92,400,638	91,833,122	27.72	27.55	99.39	97.47	92.78	41.45
91	91,763,712	91,180,234	27.53	27.35	99.36	97.69	93.30	40.63
63	87,215,514	86,715,926	26.16	26.01	99.43	97.12	92.09	41.02
87	89,153,244	88,601,197	26.75	26.58	99.38	97.60	93.09	41.37
34	84,558,180	84,096,987	25.37	25.23	99.45	97.18	92.18	41.12
