# Supplementary material for: Unveiling Genetic Markers for Milk Yield in Xinjiang Donkeys: A Genome-Wide Association Study and Kompetitive Allele-Specific PCR-Based Approach
Source: Int J Mol Sci. 2025 Mar 25;26(7):2961. doi: 10.3390/ijms26072961 (PMC11988640; doi:10.3390/ijms26072961)
Supplement: Supplementary file 1 [file ijms-26-02961-s001.zip › Table S2.pdf]

Table S2 Statistics of coverage depth and coverage ratio

Sample	Ave-depth	Cov_ratio_1X(%)	Cov_ratio_5X(%)	Cov_ratio_10X(%)
89	9	98.41	86.38	44.57
90	10	98.54	89.84	53.16
91	10	98.46	88.21	50.06
92	9	98.37	86.94	48.27
93	9	98.39	85.59	42.21
94	9	98.41	86.84	46.08
95	9	98.44	87.33	45.24
96	9	98.34	85.63	44.57
97	10	98.52	89.36	49.6
98	9	98.45	86.17	42.7
99	9	98.49	88.26	46.55
100	9	98.56	88.29	46.06
101	9	98.3	85.73	44.6
102	10	98.64	90.74	51.91
103	9	98.45	87.05	45.53
104	9	98.52	88.4	46.54
105	10	98.47	88.04	49.53
106	9	98.35	85.91	44.64
107	9	98.49	88.15	48.56
108	9	98.41	87.22	46.45
109	9	98.47	86.99	42.64
110	9	98.47	87.61	46.28
111	10	98.59	89.88	51.18
112	9	98.6	89.57	48.44
89	9	98.47	87.19	44
90	10	98.58	89.38	49.89
91	9	98.54	88.14	46.38
92	10	98.66	91.19	54.5
93	10	98.58	88.85	49.71
94	9	98.6	89.6	47.96
95	9	98.62	88.99	45.9
96	9	98.53	88.45	45.64
97	9	98.55	88.59	46.27
98	10	98.57	90.51	52.7
99	9	98.51	88.5	46.03
100	9	98.48	86.6	42.36
101	9	98.6	88.95	45.77
102	9	98.52	88.37	47.07
103	9	98.62	89.25	46.05
104	9	98.62	89.16	46.12

105	9	98.61	88.85	45.09
106	10	98.62	90.23	50.59
107	9	98.58	88.68	46.17
108	9	98.5	86.99	41.84
109	9	98.44	86.59	43.74
110	9	98.58	89.04	46.78
111	10	98.57	89.44	49.54
112	9	98.53	87.24	43.05
89	9	98.55	88.48	46.93
90	9	98.49	88	45.63
91	11	98.62	92.35	59
92	10	98.59	89.71	50.06
93	12	98.75	94.72	70.31
94	9	98.47	87.4	45.07
95	9	98.51	88.1	45.57
96	9	98.51	87.42	44.54
97	9	98.53	87.94	46.52
98	9	98.46	86.71	42.74
99	9	98.48	86.58	42.57
100	9	98.45	87.7	47.1
101	10	98.61	91.46	57.08
102	10	98.59	91.09	57.07
103	10	98.5	89.49	53.74
104	9	98.35	86.21	44.64
105	9	98.52	87.91	45.91
106	10	98.45	88.39	49.35
107	9	98.48	88.34	48.72
108	9	98.41	86.52	44.71
109	9	98.39	87.26	47.07
110	9	98.33	85.5	44.25
111	10	98.44	89.28	52.84
112	10	98.46	88.3	49.93
89	9	98.46	87.58	47.76
90	10	98.56	89.78	50.31
91	9	98.55	87.8	43.81
92	9	98.47	87.9	45.74
93	9	98.5	87.74	44.47
94	9	98.43	87.24	47.5
95	9	98.45	86.81	44.13
96	9	98.46	87.42	46.59
97	9	98.45	86.93	44.3
98	9	98.31	84.57	42.3
99	9	98.42	87	46.91
100	9	98.38	86.11	44.53

101	10	98.57	90.75	52.85
102	10	98.6	90.62	54.87
103	9	98.44	87.88	45.86
104	9	98.39	84.76	45.05
105	9	98.59	89.31	47.85
106	9	98.48	87.15	44.38
107	9	98.35	85.67	44.01
108	10	98.49	88.42	49.4
109	9	98.39	86.04	43.7
110	9	98.42	86.34	44.68
111	10	98.56	90.77	56.59
112	9	98.28	84.7	43.54
89	10	98.58	90.14	52.36
90	10	98.6	90.92	54.55
91	10	98.43	88.6	49.83
92	9	98.48	87.05	45.25
93	9	98.49	86.55	42.99
94	10	98.61	91.16	57.03
95	9	98.33	85.71	44.75
96	9	98.48	86.99	44.04
97	9	98.47	87.99	45.83
98	9	98.34	85.59	44.31
99	10	98.56	90.39	56.38
100	9	98.31	85.54	44.69
101	9	98.54	88.74	47.76
102	9	98.46	85.88	41.94
103	9	98.45	87.88	46.3
104	10	98.59	89.52	50.79
