# Supplementary material for: Unveiling Genetic Markers for Milk Yield in Xinjiang Donkeys: A Genome-Wide Association Study and Kompetitive Allele-Specific PCR-Based Approach
Source: Int J Mol Sci. 2025 Mar 25;26(7):2961. doi: 10.3390/ijms26072961 (PMC11988640; doi:10.3390/ijms26072961)
Supplement: Supplementary file 1 [file ijms-26-02961-s001.zip › Table S3.pdf]

Table S3 Statistics of comparison result

| Sample | Reads | Mapped (%) | Properly mapped (%) |
|---|---|---|---|
| 89 | 171073244 | 170545049(99.69%) | 164656522(96.25%) |
| 90 | 188988536 | 188386500(99.68%) | 181914952(96.26%) |
| 91 | 182360468 | 181841412(99.72%) | 176187066(96.61%) |
| 92 | 178814508 | 178287424(99.71%) | 172519696(96.48%) |
| 93 | 168333730 | 166481801(98.90%) | 160060282(95.09%) |
| 94 | 173409684 | 172870888(99.69%) | 167085060(96.35%) |
| 95 | 172290474 | 171778658(99.70%) | 166059870(96.38%) |
| 96 | 171728818 | 171199614(99.69%) | 165719862(96.50%) |
| 97 | 181479318 | 180850008(99.65%) | 174847004(96.35%) |
| 98 | 168330842 | 167857761(99.72%) | 162303392(96.42%) |
| 99 | 175156466 | 174621967(99.69%) | 168714736(96.32%) |
| 100 | 173686952 | 173123087(99.68%) | 166940302(96.12%) |
| 101 | 170680666 | 170164650(99.70%) | 164830382(96.57%) |
| 102 | 186405440 | 185780861(99.66%) | 178292720(95.65%) |
| 103 | 175163936 | 174628746(99.69%) | 168507700(96.20%) |
| 104 | 175964222 | 175447210(99.71%) | 169427278(96.29%) |
| 105 | 179959592 | 179419397(99.70%) | 173748174(96.55%) |
| 106 | 171609630 | 171057104(99.68%) | 165578586(96.49%) |
| 107 | 178926654 | 178372701(99.69%) | 172947370(96.66%) |
| 108 | 175799964 | 175264938(99.70%) | 169676688(96.52%) |
| 109 | 168679510 | 168156583(99.69%) | 162328646(96.23%) |
| 110 | 174959176 | 174412500(99.69%) | 168516914(96.32%) |
| 111 | 185549896 | 184956369(99.68%) | 178742998(96.33%) |
| 112 | 178582460 | 178032292(99.69%) | 172108766(96.37%) |
| 1 | 170554048 | 170040678(99.70%) | 163900128(96.10%) |
| 2 | 182393944 | 181830056(99.69%) | 175628304(96.29%) |
| 3 | 175640224 | 175145227(99.72%) | 168980872(96.21%) |
| 4 | 194353124 | 193785352(99.71%) | 186206934(95.81%) |
| 5 | 188132368 | 187589416(99.71%) | 179968718(95.66%) |
| 6 | 177115530 | 176580700(99.70%) | 170184018(96.09%) |
| 7 | 177822294 | 177328195(99.72%) | 170266420(95.75%) |
| 8 | 174698058 | 174153086(99.69%) | 167992488(96.16%) |
| 9 | 174770296 | 174212577(99.68%) | 168050292(96.15%) |
| 10 | 189986666 | 189442359(99.71%) | 182175562(95.89%) |
| 11 | 175245862 | 174696617(99.69%) | 167745022(95.72%) |
| 12 | 166363718 | 165833750(99.68%) | 160202986(96.30%) |
| 13 | 175657480 | 175136868(99.70%) | 168303162(95.81%) |
| 14 | 176357478 | 175834674(99.70%) | 170113122(96.46%) |
| 15 | 174361602 | 173799856(99.68%) | 167430464(96.02%) |
| 16 | 173686486 | 173140692(99.69%) | 167122946(96.22%) |
| 17 | 172797294 | 172238937(99.68%) | 165855304(95.98%) |
| 18 | 184888924 | 184343519(99.71%) | 177634722(96.08%) |

| | | | |
|---|---|---|---|
| 19 | 174711354 | 174141799(99.67%) | 167769836(96.03%) |
| 20 | 167037622 | 166530282(99.70%) | 160445458(96.05%) |
| 21 | 168649776 | 168115149(99.68%) | 162565416(96.39%) |
| 22 | 175509328 | 174952799(99.68%) | 168809476(96.18%) |
| 23 | 181087136 | 180537602(99.70%) | 174184126(96.19%) |
| 24 | 169282012 | 168740648(99.68%) | 162556404(96.03%) |
| 25 | 176909374 | 176338073(99.68%) | 170037010(96.12%) |
| 26 | 172437308 | 171869561(99.67%) | 166097538(96.32%) |
| 27 | 201268320 | 200633579(99.68%) | 193638012(96.21%) |
| 28 | 182579078 | 182039315(99.70%) | 175481648(96.11%) |
| 29 | 232900960 | 232189122(99.69%) | 224104630(96.22%) |
| 30 | 171966122 | 171434148(99.69%) | 165958704(96.51%) |
| 31 | 173607070 | 173046923(99.68%) | 166532454(95.92%) |
| 32 | 171903232 | 171389571(99.70%) | 165579686(96.32%) |
| 33 | 174809518 | 174259577(99.69%) | 168620940(96.46%) |
| 34 | 168193974 | 167688333(99.70%) | 162326934(96.51%) |
| 35 | 166348390 | 165764724(99.65%) | 160099106(96.24%) |
| 36 | 176327558 | 175762100(99.68%) | 169970950(96.40%) |
| 37 | 196667522 | 196103674(99.71%) | 190047406(96.63%) |
| 38 | 198367484 | 197814308(99.72%) | 191656620(96.62%) |
| 39 | 192242592 | 191630016(99.68%) | 185872962(96.69%) |
| 40 | 171496190 | 170963689(99.69%) | 165751812(96.65%) |
| 41 | 174589360 | 174062209(99.70%) | 167868198(96.15%) |
| 42 | 180354284 | 179840589(99.72%) | 174627708(96.82%) |
| 43 | 178380732 | 177787788(99.67%) | 171518002(96.15%) |
| 44 | 170881896 | 170349918(99.69%) | 164999034(96.56%) |
| 45 | 174496234 | 173935286(99.68%) | 168664180(96.66%) |
| 46 | 169257200 | 168766876(99.71%) | 163522588(96.61%) |
| 47 | 187009736 | 186430993(99.69%) | 180620442(96.58%) |
| 48 | 183347100 | 182774985(99.69%) | 177181352(96.64%) |
| 49 | 177440496 | 176903457(99.70%) | 171006944(96.37%) |
| 50 | 180447062 | 179856663(99.67%) | 173731522(96.28%) |
| 51 | 171587872 | 171075039(99.70%) | 164448496(95.84%) |
| 52 | 172988810 | 172468811(99.70%) | 166341808(96.16%) |
| 53 | 171571768 | 171038102(99.69%) | 164973828(96.15%) |
| 54 | 177470026 | 176951208(99.71%) | 171436560(96.60%) |
| 55 | 171346160 | 170845566(99.71%) | 164946546(96.27%) |
| 56 | 175812276 | 175291592(99.70%) | 169596316(96.46%) |
| 57 | 169812328 | 169284264(99.69%) | 163136912(96.07%) |
| 58 | 165939584 | 165443467(99.70%) | 160059064(96.46%) |
| 59 | 174175700 | 173659329(99.70%) | 168511736(96.75%) |
| 60 | 171146614 | 170664322(99.72%) | 165489964(96.69%) |
| 61 | 187423684 | 186856247(99.70%) | 180421290(96.26%) |
| 62 | 195398398 | 194821528(99.70%) | 186816594(95.61%) |

| | | | |
|---|---|---|---|
| 63 | 173431852 | 172887723(99.69%) | 167184242(96.40%) |
| 64 | 182036932 | 181505709(99.71%) | 173018596(95.05%) |
| 65 | 180276310 | 179727264(99.70%) | 172777448(95.84%) |
| 66 | 170961392 | 170423091(99.69%) | 164492694(96.22%) |
| 67 | 169305134 | 168735682(99.66%) | 163290362(96.45%) |
| 68 | 181058306 | 180484438(99.68%) | 174161072(96.19%) |
| 69 | 170248958 | 169751854(99.71%) | 163868058(96.25%) |
| 70 | 172595124 | 172076151(99.70%) | 166519568(96.48%) |
| 71 | 197179840 | 196567118(99.69%) | 189516280(96.11%) |
| 72 | 168018820 | 167543563(99.72%) | 162718214(96.85%) |
| 73 | 188071110 | 187507833(99.70%) | 180737970(96.10%) |
| 74 | 191642878 | 191078838(99.71%) | 184199560(96.12%) |
| 75 | 182369882 | 181849202(99.71%) | 175600522(96.29%) |
| 76 | 172095080 | 171587371(99.70%) | 165758960(96.32%) |
| 77 | 170550126 | 170053460(99.71%) | 163453706(95.84%) |
| 78 | 199932648 | 199351041(99.71%) | 192572378(96.32%) |
| 79 | 170580926 | 170087557(99.71%) | 164734702(96.57%) |
| 80 | 170021000 | 169520937(99.71%) | 163880230(96.39%) |
| 81 | 173611754 | 173106488(99.71%) | 166904950(96.14%) |
| 82 | 170244522 | 169751041(99.71%) | 164374114(96.55%) |
| 83 | 197858378 | 197252556(99.69%) | 190634894(96.35%) |
| 84 | 170327920 | 169790170(99.68%) | 164481728(96.57%) |
| 85 | 179834876 | 179322344(99.71%) | 172597336(95.98%) |
| 86 | 167043808 | 166569706(99.72%) | 160749186(96.23%) |
| 87 | 177202394 | 176698827(99.72%) | 170338412(96.13%) |
| 88 | 183666244 | 183120510(99.70%) | 176613860(96.16%) |
