# Supplementary material for: Unveiling Genetic Markers for Milk Yield in Xinjiang Donkeys: A Genome-Wide Association Study and Kompetitive Allele-Specific PCR-Based Approach
Source: Int J Mol Sci. 2025 Mar 25;26(7):2961. doi: 10.3390/ijms26072961 (PMC11988640; doi:10.3390/ijms26072961)
Supplement: Supplementary file 1 [file ijms-26-02961-s001.zip › Table S4.pdf]

Table S4 Relationship between SNPs and genes

Type	SNP number
Intergenic	5,201,198
Intron	4,738,320
Upstream	898,713
Downstream	730,993
UTR_3_prime	118,007
Intragenic	91,113
CDS synonymous coding	79,991
CDS non synonymous coding	74013
Exon	53,095
UTR_5_prime	42,707
Start gained	8,878
CDS Stop gained	1,128
Splice site donor	684
Splice site acceptor	584
Start lost	204
Stop lost	172
Synonymous stop	69
Non synonymous start	40
